# The molecular mechanism of anticancer action of novel octahydropyrazino[2,1-a:5,4-a′]diisoquinoline derivatives in human gastric cancer cells

**DOI:** 10.1007/s10637-018-0584-y

**Published:** 2018-03-17

**Authors:** Natalia Pawłowska, Agnieszka Gornowicz, Anna Bielawska, Arkadiusz Surażyński, Anna Szymanowska, Robert Czarnomysy, Krzysztof Bielawski

**Affiliations:** 1 Department of Synthesis and Technology of Drugs, Medical University of Bialystok, 15-222 Bialystok, Poland; 2 Department of Biotechnology, Medical University of Bialystok, Kilinskiego 1, 15-222 Bialystok, Poland; 3 Department of Medicinal Chemistry, Medical University of Bialystok, 15-222 Bialystok, Poland

**Keywords:** AGS-CRL-1739 cells, Gastric cancer, Apoptosis, Topo II inhibitors, Cell signaling, Diisoquinoline alkaloids

## Abstract

*Objective* The aim of the current study was to examine the anticancer activity and the detailed mechanism of novel diisoquinoline derivatives in human gastric cancer cells (AGS). *Methods* The viability of AGS cells was measured by MTT (3-(4,5-dimethylthiazol-2-yl)-2,5-diphenyltetrazolium bromide) assay. Cell cycle analysis and apoptosis assay were performed by standard flow cytometric method. Confocal microscopy bioimaging was used to demonstrate the expression of pivotal proteins engaged in apoptosis (caspase-8, caspase-3, p53) and cell signaling (AKT, ERK1/2). *Results* All compounds decreased the number of viable cells in a dose-dependent manner after 24 and 48 h of incubation, although compound 2 was a more cytotoxic agent, with IC_50_ values of 21 ± 2 and 6 ± 2 μM, compared to 80 ± 2 and 45 ± 2 μM for etoposide. The cytotoxic and antiproliferative effects of novel compounds were associated with the induction of apoptosis. The highest percentage of early and late apoptotic cells was observed after 48 h of incubation with compound **2** (89.9%). The value was higher compared to compound 1 (20.4%) and etoposide (24.1%). The novel diisoquinoline derivatives decreased the expression of AKT and ERK1/2. Their mechanism was associated with p53-mediated apoptosis, accumulation of cells in the G2/M phase of cell cycle and inhibition of topoisomerase II. *Conclusion* These data strongly support compound 2 as a promising molecule for treatment of gastric cancer.

## Introduction

Gastric cancer (GC) is a multifactorial disease and still rates as the second most common cause of cancer related deaths in the world [1]. Dietary habits, environmental influences and infections to underlying epigenetics factors are the main contributors to the incidence of the disease [2]. Among the predisposing factors are: *Helicobacter pylori* infection, high salt intake and smoking, which strongly increase the risk of gastric cancer [3]. Insufficient effectiveness of chemotherapy and lack of reliable markers to predict the response to chemotherapy in gastric cancer are associated with high mortality [4]. Data show that 50% of advanced GC patients suffer from local or systemic recurrence even after standard adjuvant treatment, and only 10–15% of all GC patients achieve 5-year overall survival [5, 6] There is still a need to look for novel chemotherapeutic agents, more active then those commonly used in gastric cancer treatment.

Recently our team has synthesized a group of novel octahydropyrazino[2,1-a:5,4-a′]diisoquinoline derivatives. We evaluated their cytotoxic activity and antiproliferative potency in MCF-7 and MDA-MB-231 breast cancer cell lines. We observed that all compounds induced apoptosis. We demonstrated higher activity of caspases 3, 8, 9 and 10, which confirmed that the induction of apoptosis is associated with external and internal cell death pathway. Our study revealed that the novel compounds in the group of diisoquinoline derivatives are promising candidates in anticancer treatment by activation of both extrinsic and intrinsic apoptotic pathways [7].

The aim of this study was to check the anticancer activity and the detailed mechanism of the most active diisoquinoline derivatives in human gastric cancer cells (AGS). After preliminary study, the most cytotoxic agents (**1** and **2**) were selected for further investigations. Their anticancer potential was compared with etoposide, which is a commonly known chemotherapeutic agent in gastric cancer treatment. The effect of the tested compounds (**1**, **2**, etoposide) on viability, DNA biosynthesis and cell cycle in AGS cells was investigated. Electrophoresis was performed to prove that the compounds are topoisomerase II inhibitors. Annexin V binding assay and dual acridine orange/ethidium bromide staining were used to confirm apoptosis induction. Bioimaging was applied as a tool to explain in detail the molecular mechanism of the compounds tested. The expressions of pivotal proteins involved in apoptosis and cell signaling, such as initiator and effector caspases: −9 and 3, p53, AKT, ERK1/2 were analyzed.

## Materials and methods

### Chemicals and consumables

Methanol and ethidium bromide,3-(4,5-dimethylthiazol-2-yl)-2,5-diphenyltetrazolium bromide (MTT), were purchased from Sigma Chemical Co. (USA). Stock cultures of AGS- CRL-1739 human stomach cancer cells were purchased from the American Type Culture Collection (USA). Ham’s F-12 K (Kaighn’s) Medium and fetal bovine serum (FBS) used in a cell culture were products of Gibco (USA). Glutamine, penicillin and streptomycin were obtained from Quality Biologicals Inc. (USA). [^3^H]thymidine (6.7 Ci mmol^−1^) was purchased from NEN (USA), and Scintillation Coctail “Ultima Gold XR” from Packard (USA). Sodium dodecyl sulfate was received from Bio-Rad Laboratories (USA). Acridine orange and ethidium bromide were provided by Sigma Chemical Co (USA). FITC Annexin V Apoptosis Detection Kit II was a product of BD Pharmigen. Topoisomerase II Drug Screening Kit was a product of TopoGEN (Florida, USA).

### Compounds

The octahydropyrazin[2,1-a:5,4-a′]diisoquinoline derivatives (**1, 2**) were synthesized using previously standardized methods [7–9].

### Cell culture

AGS human gastric adenocarcinoma cells were maintained in a base growth medium – F-12 K, supplemented with fetal bovine serum (FBS) to a final concentration of 10% and 1% antibiotics (penicillin/streptomycin). Cells were cultured in Costar flasks and grown in 5% CO_2_ at 37 °C in high humid atmosphere to subconfluence (90–95%). Subconfluent cells were treated with 0.05% trypsin and 0.02% EDTA in calcium free phosphate buffered saline, counted in hemocytometer and seeded at 5 × 10^5^ cells/well in 6-well plates (Nunc) in 2 mL of the growth medium (F-12 K). Cells which reached about 80% of confluency were used in the present study.

### Cell viability assay

Cell growth was assessed in AGS cells following treatment with the compounds tested using MTT (3-(4,5-dimethylthiazole-2-yl)-2,5-diphenyltetrazolium bromide), according to the method of Carmichael et al. [10]. The cells were incubated with different concentrations of compounds **1**, **2** and etoposide for 24 and 48 h to determine the IC_50_ value for each drug. Confluent cells, treated for 24 and 48 h with the compounds in 6-well plates, were washed three times with PBS and then incubated for 4 h in 1 mL of MTT solution (5 mg/mL of stock in PBS) at 37 °C in the atmosphere of 5% CO_2_ in an incubator. Then, supernatant was discarded and 1 mL of 0.1 mol/L HCl in absolute isopropanol was added to each well and mixed gently. The absorbance of converted dye in living cells was read at a wavelength of 570 nm using spectrophotometer (Helios Gamma U*V*/VIS Scanning Spectrophotometer, Unicam/ThermoFisher Scientific Inc., Waltham, MA, USA). Untreated cells were also run under identical conditions and served as control. The viability of stomach cancer cells cultured in the presence of the compounds tested was calculated as a percent of control cells.

### [^3^H]thymidine incorporation assay

The incorporation of [^3^H]thymidine into DNA was used as a measure of cell proliferation. AGS cells were seeded in 6-well plates at a density of 5 × 10^5^ well^−1^ in a complete growth medium and grown as described above. The cells were incubated for 24 and 48 h with various concentrations of compounds **1**, **2** and etoposide in 5% CO_2_ at 37 °C before 0.5 μCi of [^3^H]thymidine was added to each well for 4 h to measure the incorporation of radioactive component into DNA**.** Then, the cells were harvested by trypsinisation and washed several times with cold phosphate buffered saline (10 min/1500 g) until the dpm in the washes were similar to the control reagent. Radioactivity was determined in a scintillation counter (Packard Tri-Carb Liquid Scintillation Counter 1900 TR, Perkin Elmer, Inc.,Waltham, MA, USA). [^3^H]thymidine uptake was expressed as dpm well^−1^.

### Cell cycle analysis

The distribution of the cell cycle phases was analyzed by flow cytometry. Briefly, AGS-CRL-1739 human gastric cancer cells were seeded into 6-well plates at a density of 2.5 × 10^5^ cells per well and treated with 20 μM of compounds **1**, **2** and etoposide for 24 and 48 h. After incubation, the cells were harvested and then fixed with 1 mL of 70% ethanol and kept overnight at −20 °C. Before analysis, the cells were resuspended in PBS, treated with 50 μg/mL of DNase-free RNase A Solution (Promega), and stained with 100 μg/mL of PI. The FACSCanto II flow cytometer (BD Bioscences Systems, San Jose, CA, USA) was used to read the fluorescence.

### Relaxation assay of topo II

Supercoiled pHOT DNA (0.5 mg) was incubated with 4 units of human topoisomerase II in the cleavage buffer (30 mM Tris–HCl (pH 7.8), 50 mM KCl, 10 mM MgCl_2_, 3 mM ATP, 15 mM mercaptoethanol), in the presence of the compounds tested (100 μM). Reactions were carried out at 37 °C for 1 h and then terminated by the addition of 2 mL of 10% SDS and 2 mL of 50 μg/mL proteinase K. The reaction mixture was subjected to electrophoresis through a 0.8% agarose gel containing 0.5 mg/mL ethidium bromide in TBE buffer (90 mM Tris–borate and 2 mM EDTA). The gels were stained with ethidium bromide and photographed under UV light.

### Flow cytometry assessment of Annexin V binding

The effect on the induction of apoptosis after 24 and 48 h of incubation with the compounds tested was determined by Becton Dickinson FACSCanto II flow cytometer FACSCanto II (Becton Dickinson Bioscences Systems, San Jose, CA, USA), assessing the loss of asymmetry of the phospholipids on the cell membrane. Cells were trypsinised, resuspended in F12-K and then in binding buffer. Next, they were stained with FITC Annexin V and propidium iodide (PI) for 15 min at room temperature in the dark according to the manufacturer’s instruction (FITC Annexin V Apoptosis Detection Kit II). Cells cultured in a drug-free medium were used as controls. Optimal parameter settings were found using a positive control (cells incubated with 3% formaldehyde in buffer during 30 min on ice). Forward scatter (FS) and side scatter (SC) signals were detected on a logarithmic scale histogram. FITC was detected in the FL1 channel (FL1 539; Threshold–value 52). The results were analyzed with FACSDiva software (Becton Dickinson Biosciences Systems, San Jose, CA, USA).

### Dual acridine orange/ethidium bromide fluorescent staining

To confirm the rates of apoptosis, obtained using the FITC Annexin V Apoptosis Detection Kit II, we carried out an assessment of the dual acridine orange/ethidium bromide fluorescent staining, visualized under a fluorescent microscope Nikon Eclipse Ti with an inverted camera (Nikon Instruments Inc., Melville, NY, USA). AGS-CRL-1739 human gastric cancer cells were treated with the tested compounds **1**, **2** and etoposide (20 μM) for 24 and 48 h. The cell suspension (250 μl) was stained with 10 μl of the dye mixture (10 μM acridine orange and 10 μM ethidium bromide), which was prepared in PBS. Cells cultured in a drug-free medium were used as controls. The morphology of two hundred cells per sample was examined by fluorescent microscopy within 20 min. The results were analyzed with NIS-Elements software (Nikon Instruments Inc., Melville, NY, USA).

### Immunofluorescence staining and bioimaging

Cells were seeded in BD Falcon™ 96-well black, clear-bottom tissue culture plates at 10.000 cells per well. These plates are optimized for imaging applications. Analysis were performed in three independent experiments. After incubation, the cells were rinsed with PBS and fixed with a 3.7% formaldehyde solution at room temperature for 10 min. Then, they were washed three times with PBS and permeabilized with 0.1% Triton X-100 at room temperature for 5 min. Next, they were washed twice with PBS, and non-specific binding was blocked by incubation in 3% FBS at room temperature for 30 min. The cells were rinsed and incubated with anti-caspase-9 mouse monoclonal antibody (BD Biosciences, San Jose, CA, USA; 1:200), or anti-caspase-3 (Ab-2) mouse monoclonal antibody (Cell Signaling Technology, Danvers, MA, USA; 1:200), or anti- p53 (DO-1) mouse monoclonal antibody (Santa Cruz Biotechnology, Dallas, TX, USA, 1:200), or anti-p-AKT (anti-phospho-PKB (pThr^308^) rabbit polyclonal antibody (Sigma-Aldrich, St. Louis, MO, USA; 1:200) or anti-ERK1/ERK2 (pT202/pY204) mouse monoclonal antibodies (BD Biosciences, San Jose, CA, USA; 1:200) for 1 h at room temperature. Then, they were washed three times with PBS and incubated with FITC-conjugated anti-rabbit or anti-mouse secondary antibodies (BD Biosciences, San Jose, CA, USA, 1:500) for 60 min in the dark. After washing, nuclei were stained with Hoechst 33,342 (2 μg/ml, *blue*) and analyzed using a BD Pathway 855 confocal microscope with a 40 × (0.75 NA) objective. The cytoplasmic and nuclear fluorescence intensities of stained cells were analyzed, and images of FITC-labeled cells were acquired using a 488/10 excitation laser and a 515LP emission laser.

### Statistical analysis

Experimental data were presented as mean ± standard deviation (SD) since each experiment was repeated at least three times.

## Results

### Novel diisoquinoline derivatives inhibit the growth of gastric cancer cells in a dose and time-dependent manner

**Fig. 1:**
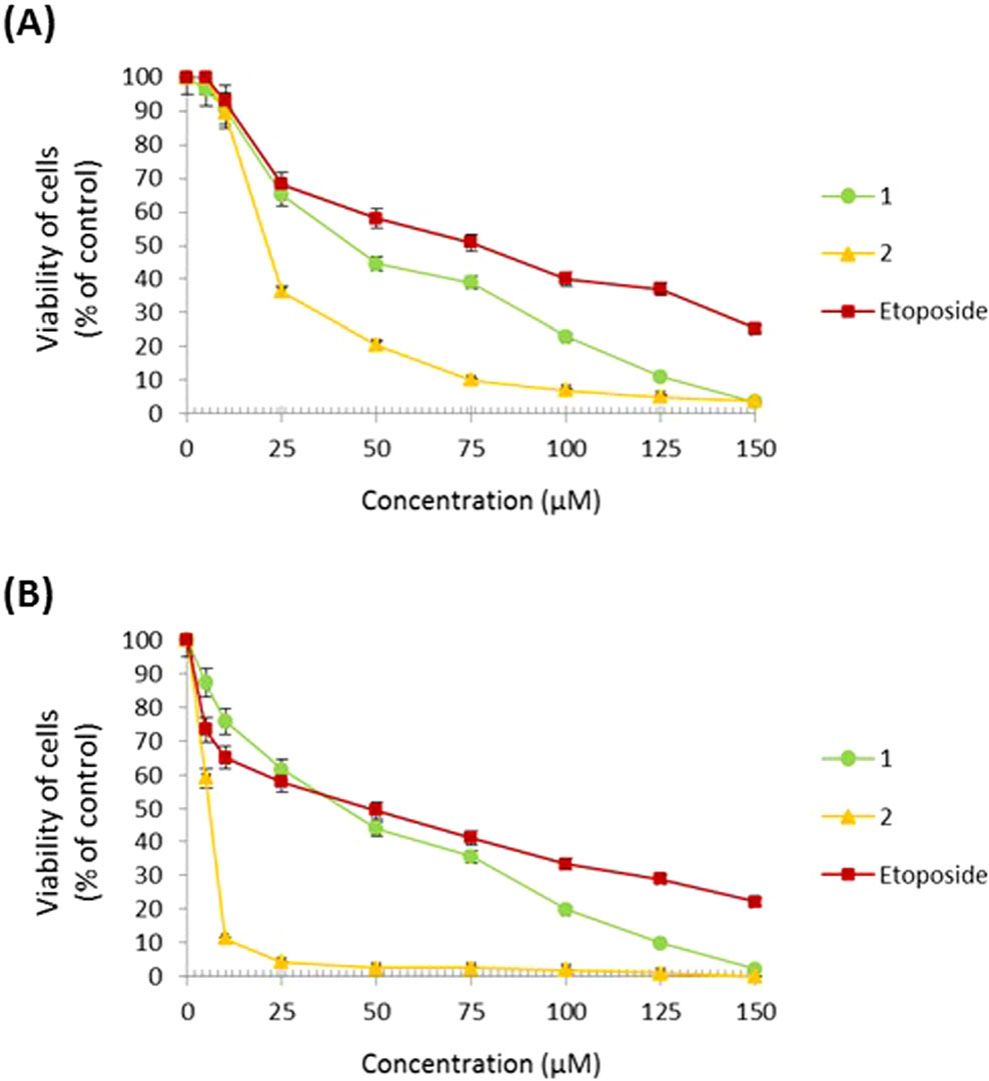
Viability of AGS cells treated for 24 h (**a**) and 48 h (**b**) with different concentrations of the tested compounds (**1, 2)** and etoposide. Mean ± SD from three independent experiments (*n* = 3) done in duplicate are presented

At the beginning, the growth inhibitory effect of the compounds **1**, **2** and etoposide on human gastric cancer cells was examined. The results revealed that all compounds exerted a time-dependent growth inhibitory effect. IC_50_ values for compound 1 were 44 μM and 40 μM at 24 and 48 h. IC_50_ values for compound 2 were 21 μM and 6 μM after 24 and 48 h of incubation, respectively. The reference compound – etoposide represented the weakest cytotoxic potential with IC_50_ values of 80 μM and 45 μM at 24 and 48 h, respectively (Fig. 1).

**Fig. 2:**
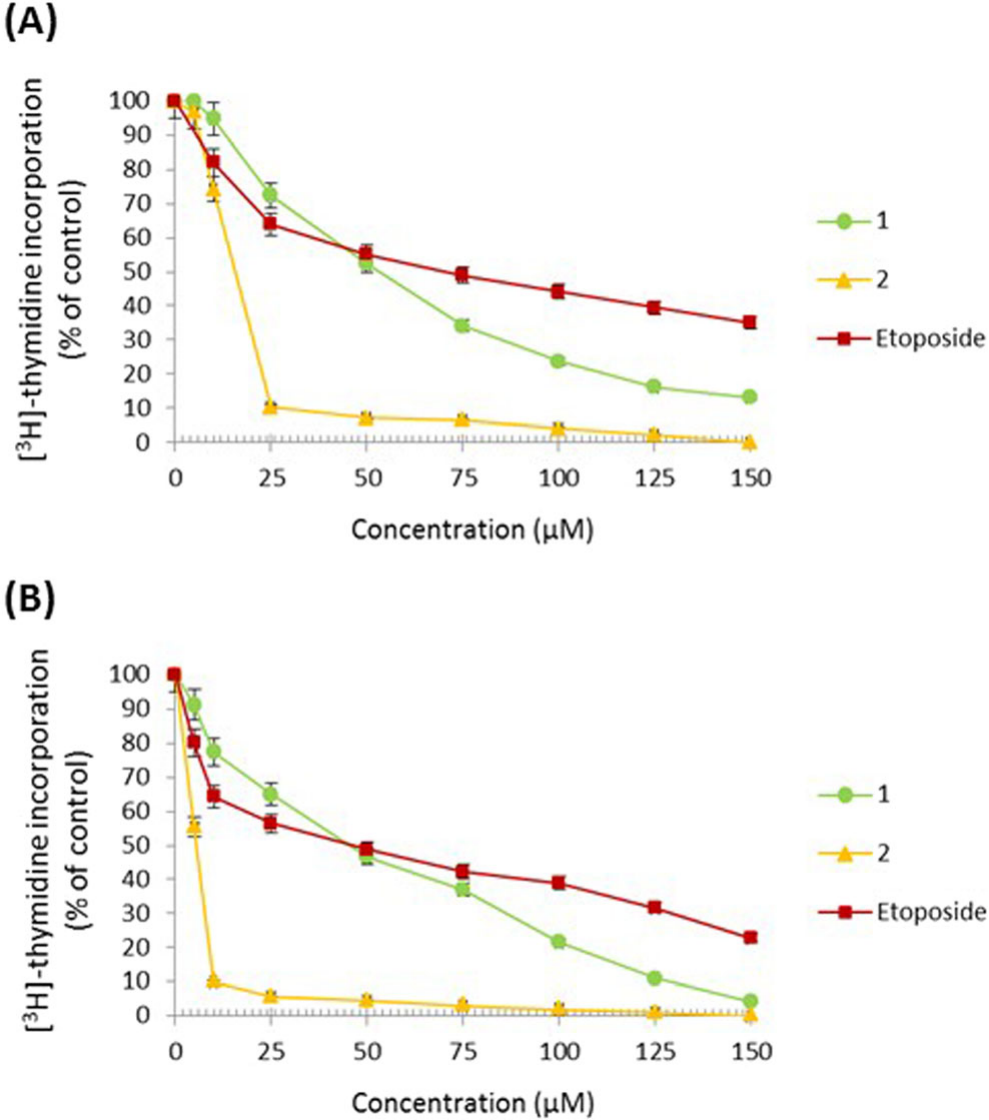
Antiproliferative effects of the tested compounds (**1**, **2**) and etoposide in cultured gastric AGS cells after 24 h (**a**) and 48 h (**b**) incubation as measured by inhibition of [^3^H]-thymidine incorporation into DNA. Mean ± SD from three independent experiments (n = 3) done in duplicate are presented

The effect of the compounds on DNA biosynthesis was also investigated. We proved that compound 2 had the strongest time-dependent antiproliferative effect with IC_50_ values of 16 μM and 6 μM at 24 and 48 h, respectively. Compound 1 at a weaker rate inhibited DNA biosynthesis in the analyzed cells with IC_50_ values of 54 μM and 44 μM at 24 and 48 h. Etoposide represented the weakest antiproliferative potential with IC_50_ values of 68 μM and 44 μM at 24 and 48 h (Fig. 2).

### Novel diisoquinoline derivatives induced G2/M gastric cancer cell cycle arrest and inhibited topoisomerase II in gastric cancer cells

**Fig. 3:**
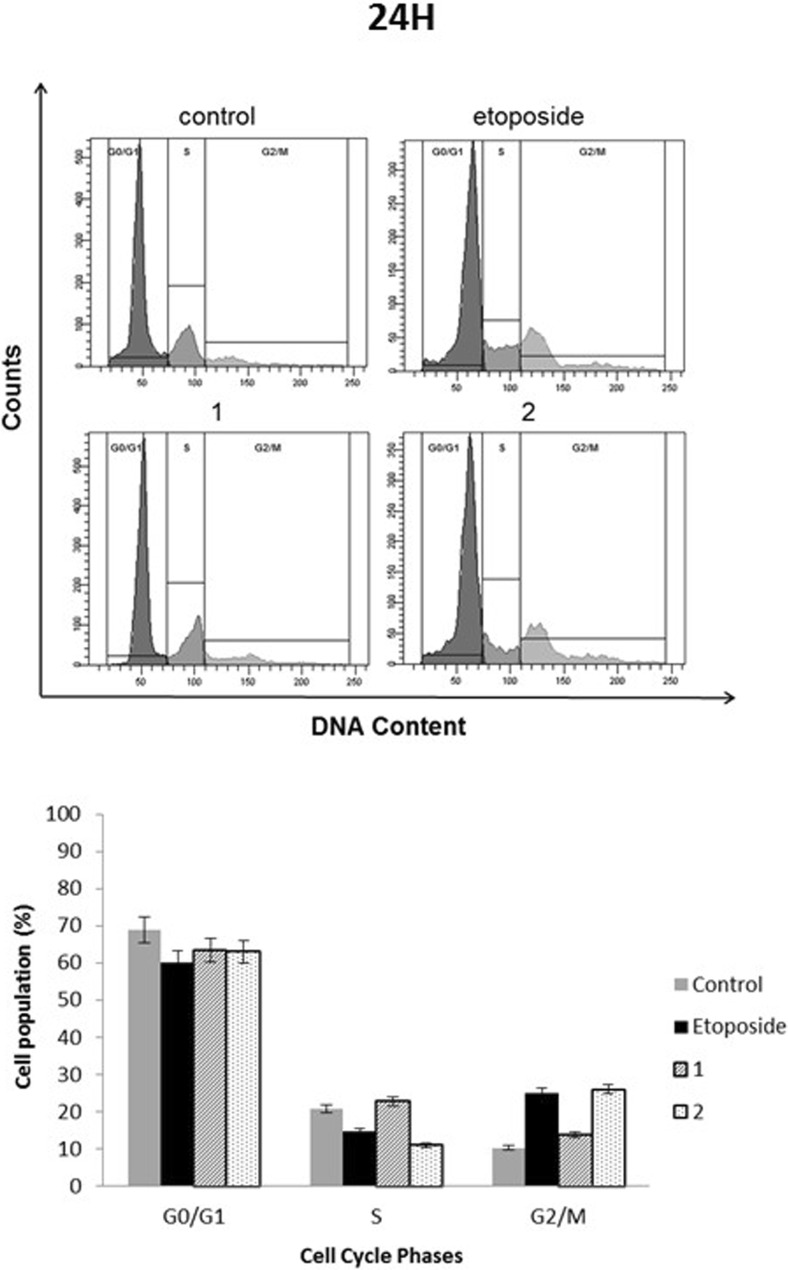
Effect of compounds **1**, **2** and etoposide (20 μM) on cell cycle distribution in human stomach AGS cells after 24 h of incubation. **a** Representative flow cytometry profiles depicting the cell cycle distribution. **b** Histograms representing the percentages of the cell population at each phase of the cell cycle. Mean ± SD from three independent experiments (n = 3) done in duplicate are presented

**Fig. 4:**
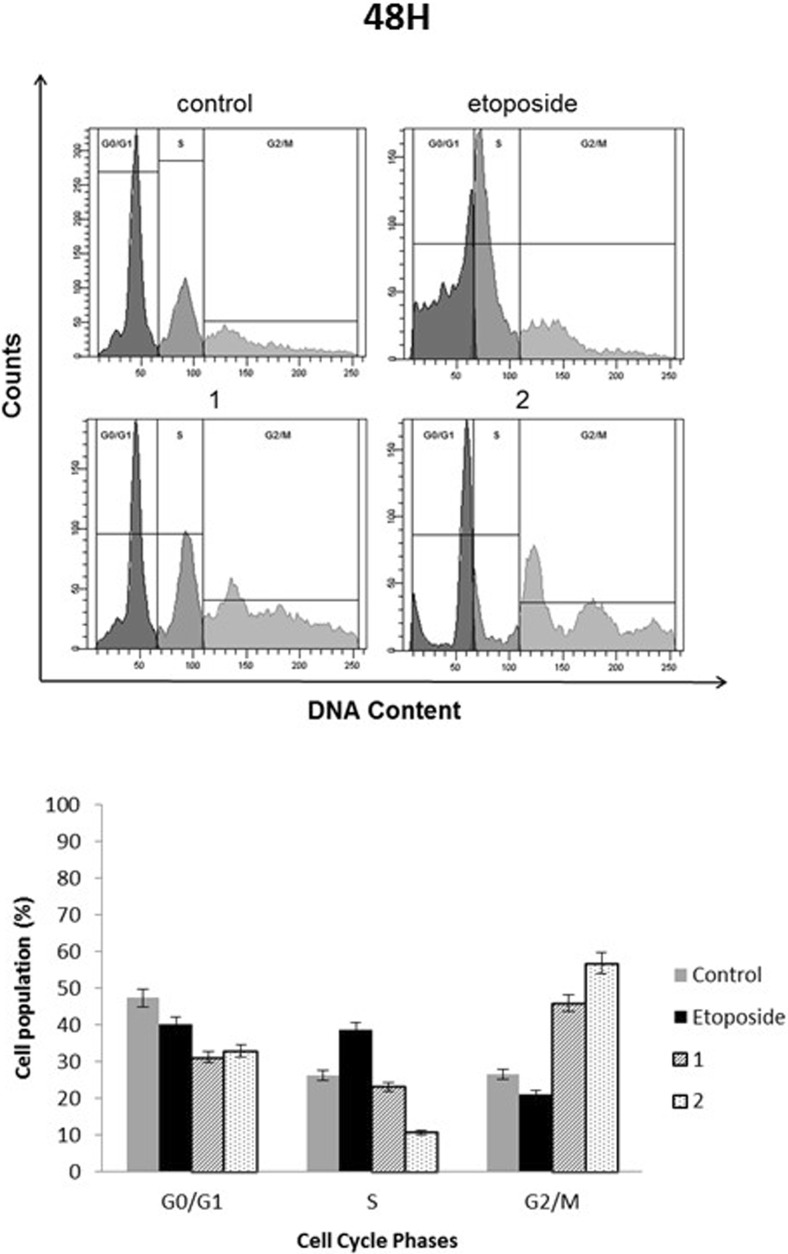
Effect of compounds **1**, **2** and etoposide (20 μM) on cell cycle distribution in human stomach AGS cells after 48 h of incubation. **a** Representative flow cytometry profiles depicting the cell cycle distribution. **b** Histograms representing the percentages of the cell population at each phase of the cell cycle. Mean ± SD from three independent experiments (n = 3) done in duplicate are presented

The literature data show that etoposide induces G2/M cell cycle arrest and its mechanism of action is associated with inhibition of topoisomerase II [11]. The effect of our novel diisoquinoline derivatives on cell cycle and topoisomerase II inhibition was examined (Figs. 3 and 4). The novel compounds led to the accumulation of cells in the G2/M phase of cell cycle. The percentage of cells in the G2/M phase increased from 10.3% in the untreated control to 13.8% after treatment with compound 1 (20 μM), 25.1% after treatment with etoposide (20 μM) and 26% after treatment with compound **2** (20 μM). Upon prolongation of the exposure time to 48 h, the highest percentage of cells were arrested in the G2/M phase after treatment with compound **2** (56.7%).

**Fig. 5:**
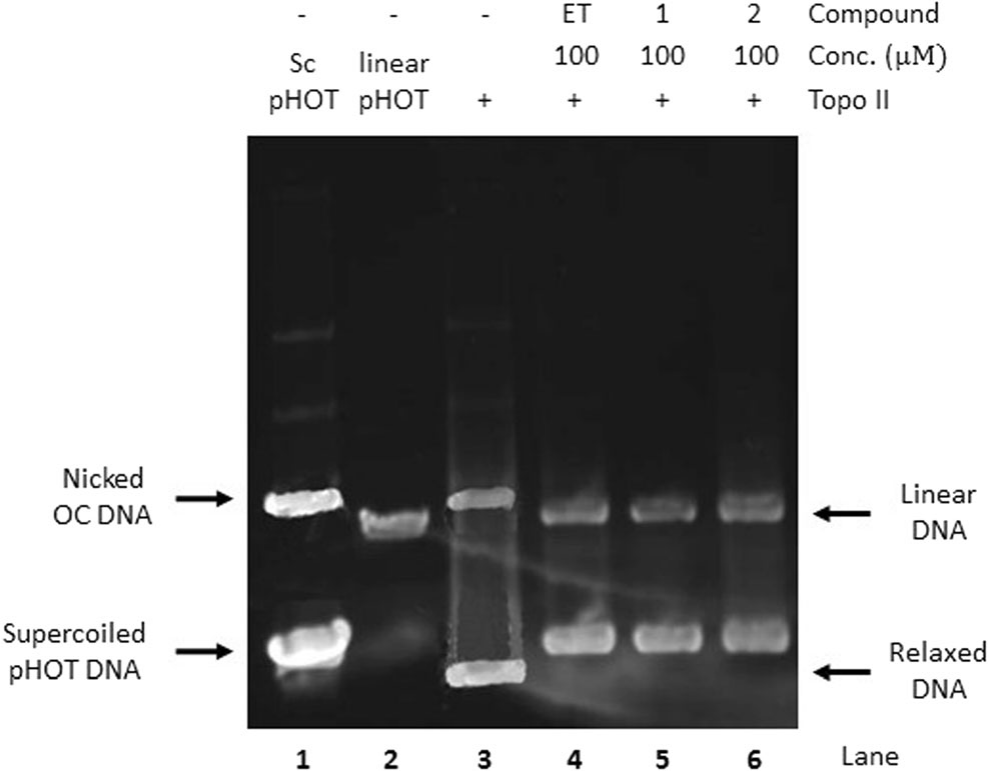
Inhibition of topoisomerase II-mediated DNA supercoiling in the presence of the tested compounds (**1, 2**) and etoposide (ET). Supercoiled pHOT DNA (lane 1) was incubated with topoisomerase II in the absence (lane 3) or in the presence of drug (lane 4–6) at the concentration of 100 μM. DNA was analyzed by 0.8% agarose gel electrophoresis. The gels were stained with ethidium bromide and photographed under UV light

The ability of the compounds **1**, **2** to inhibit topoisomerase II activity was quantified by detecting Topo II cleavage products, including linear DNA. The obtained results demonstrated that the compounds **1** and **2**, the same as a known Topo II poison etoposide (VP-16), stimulated the stabilization of the cleavable complex at the concentration of 100 μM (Fig. 5).

### Novel diisoquinoline derivatives induce apoptosis in gastric cancer cells

**Fig. 6:**
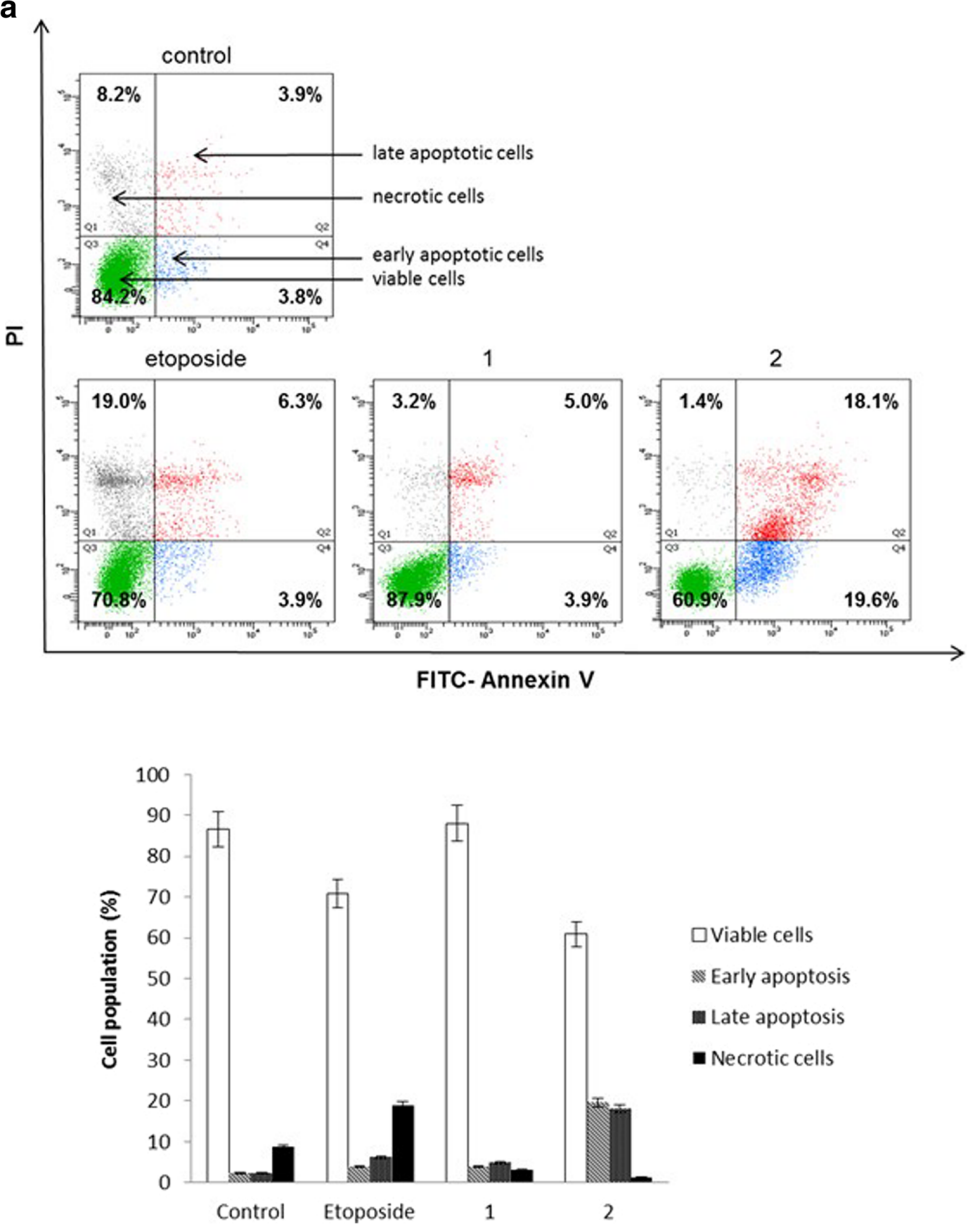

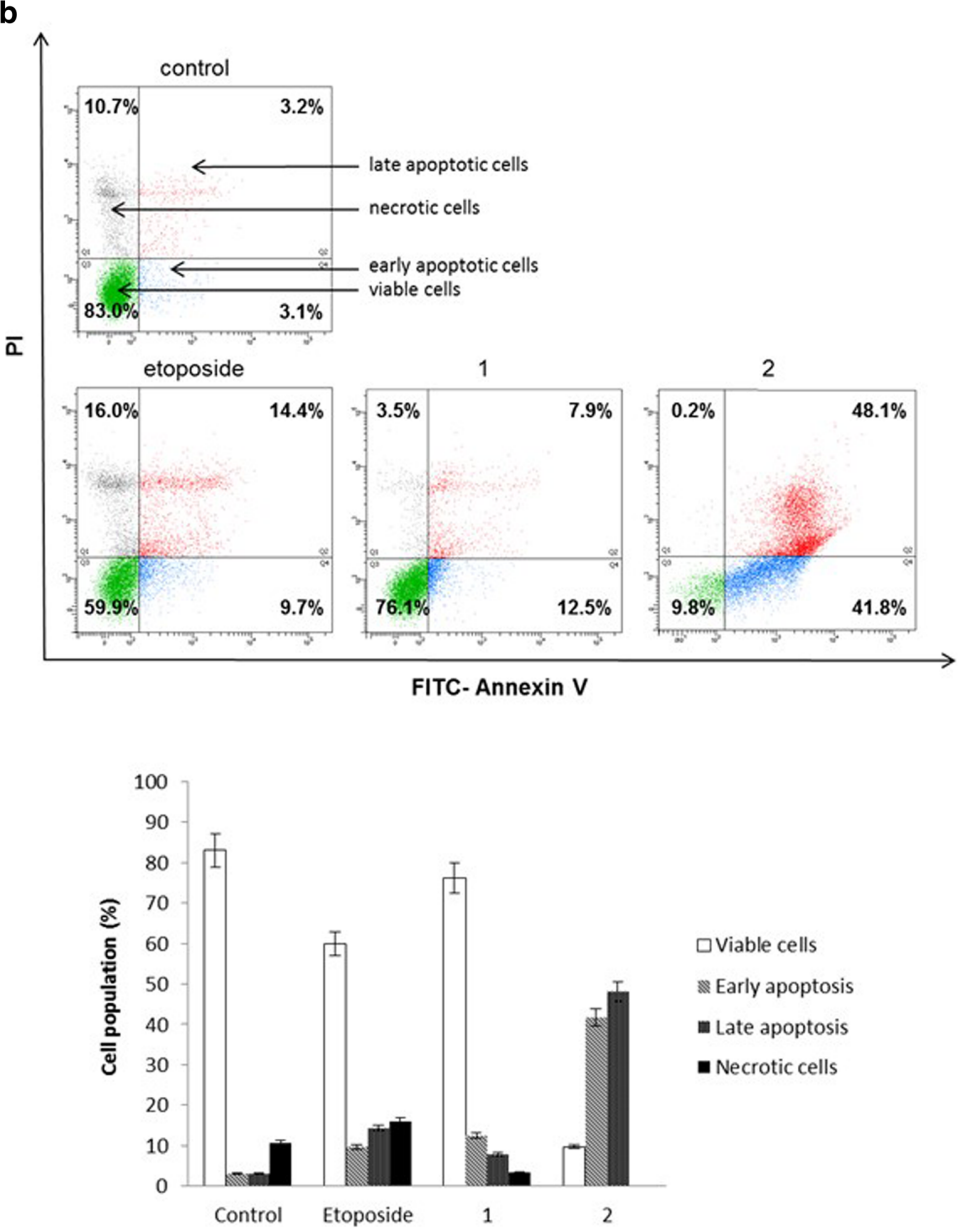
Flow cytometric analysis of AGS cells after incubation with 20 μM of the tested compounds (**1**, **2**) and etoposide for 24 h (**a**) and 48 h (**b**) and subsequent staining with annexin V and propidium iodide (PI). Dots with annexin V^−^/PI^−^ (Q3), annexin V^+^/PI^−^ (Q4), annexin V^−^/PI^−^ (Q1), and annexin V^+^/PI^+^ (Q2) feature represent intact, early apoptotic, late apoptotic, and necrotic cells, respectively. Mean percentage values from three independent experiments (n = 3) done in duplicate are presented

Annexin-V binding assay was used to detect early and late apoptotic cells (annexin-V^+^/PI^−^ and annexin-V^+^/PI^+^), necrotic (annexin-V^−^/PI^+)^ as well as viable cells (annexin-V^−^/PI^−^). The test is based on the externalization of phosphatidylserine (PS) at the outer leaflet of the plasma membrane. Annexin V-FITC possess high affinity for the PS, which is exposed to the extracellular environment during programmed cell death. Propidium iodide was used to determine necrotic cells [12]. After 24 h of incubation with compounds **1**, **2** and etoposide we observed that compound **2** strongly induced apoptosis in comparison with untreated cells. We detected 19.6% of early and 18.1% of late apoptotic cells, compared to 3.8% of early and 3.9% of late apoptotic cells in the control sample. Etoposide as well as compound 1 insignificantly increased the percentage of early and late apoptotic cells in comparison with untreated cells. The ratio of early and late apoptotic cells was increased from 7.7% to 10.2% after etoposide treatment and from 7.7% to 8.9% after compound 1 treatment (Fig. 6a). After 48 h of incubation a significant proapoptotic effect was observed in gastric cancer cells in all cases. The proportion of viable AGS cells after treatment with compound **2** decreased from 83% to 9.8%. The percentage of viable cells decreased from 83% to 59.9% after etoposide treatment and from 83% to 76.1% after compound 1 treatment (Fig. 6b). These results suggest that cytotoxicity of the compounds against AGS cancer cells is due to the induction of apoptotic cell death.

**Fig. 7:**
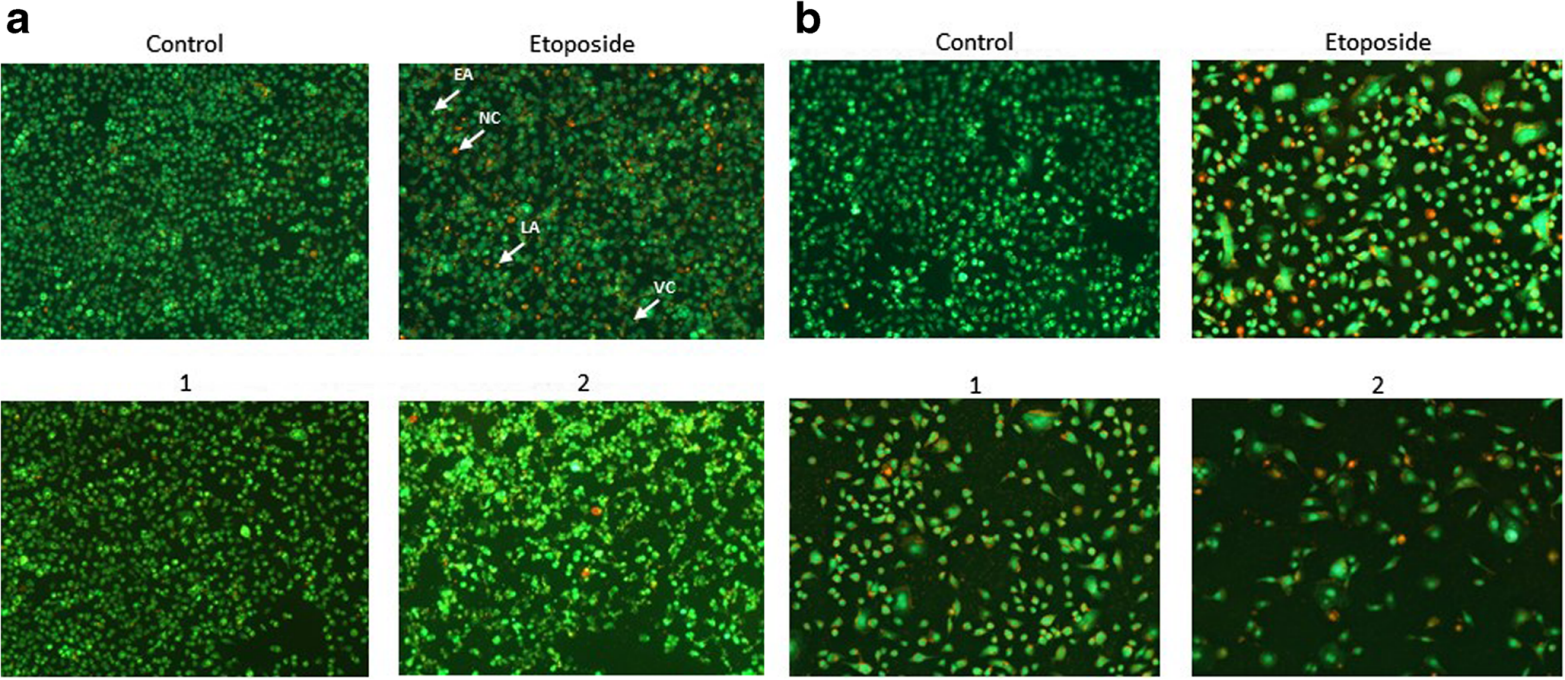
Induction of apoptosis in human stomach AGS cells treated for 24 h (**a**) and 48 h (**b**) with the compounds (**1**, **2)** and etoposide (20 μM) evaluated by a fluorescent microscopy after acridine orange and ethidium bromide staining (VC- viable cells, EA- early apoptosis, LA- late apoptosis, NC- necrotic cells)

To reveal the morphology of apoptosis in gastric cancer cells after treatment with the compounds tested, the acridine orange (AO) and propidium iodide (PI) double staining was performed. The untreated cells with normal nuclear structure were displayed as green fluorescence. The early stage of apoptosis was identified as bright green fluorescence, whereas the late apoptosis was identified by reddish-orange color. Our study proved that compound 2 possessed the strongest proapoptotic properties in comparison with untreated cells as well as with cells incubated with compound **1** and etoposide (Fig. 7a). The effect was enhanced after 48 h of incubation (Fig. 7b). In all cases, the changes in cell morphology characteristic of apoptosis, such as chromatin condensation and membrane blebbing, were observed.

### Novel diisoquinoline derivatives activated initiator and executioner caspases in AGS cells

**Fig. 8:**
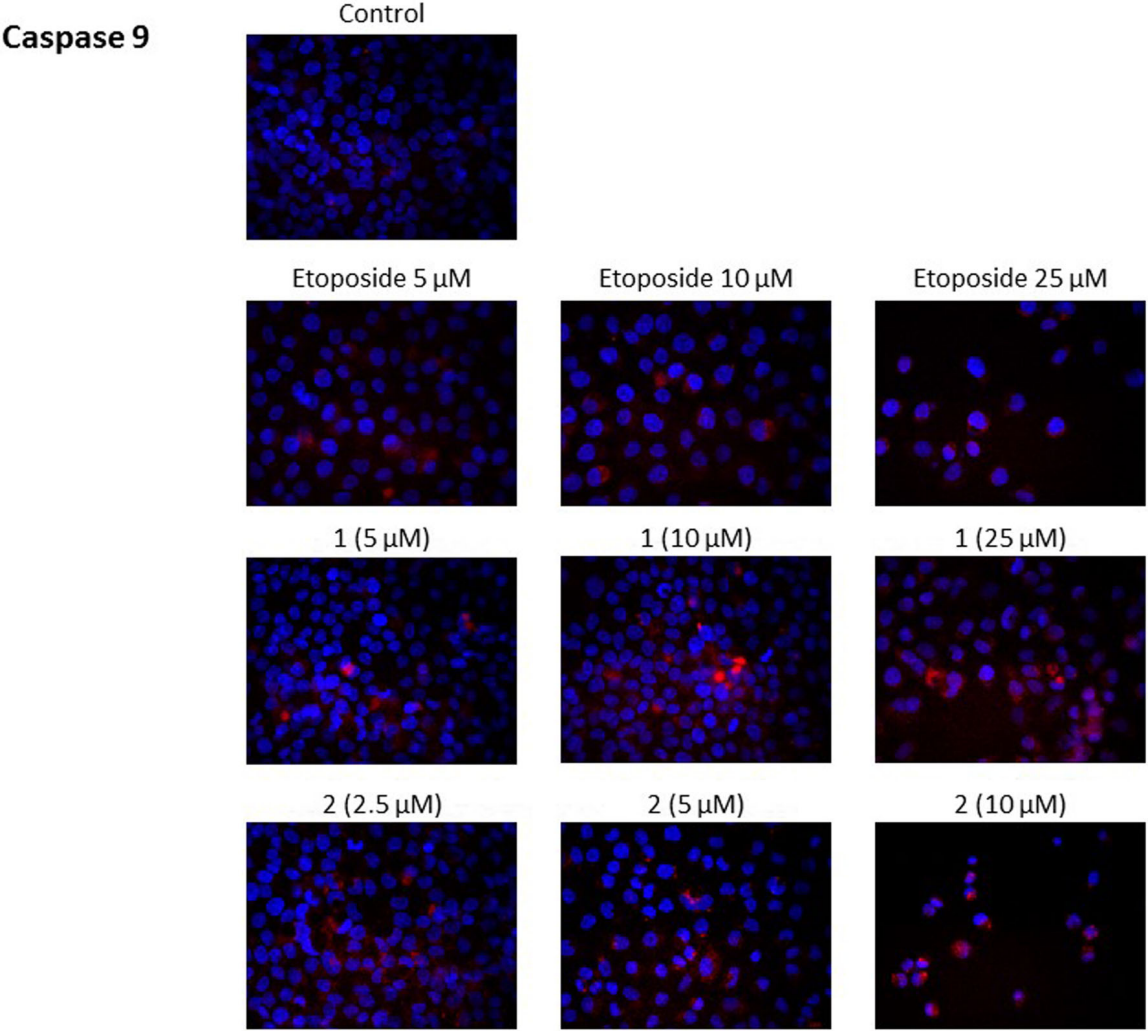
Immunofluorescence analysis of caspase 9 expression and translocation in human gastric AGS cells treated with different concentrations of the tested compounds (**1, 2**) and etoposide

**Fig. 9:**
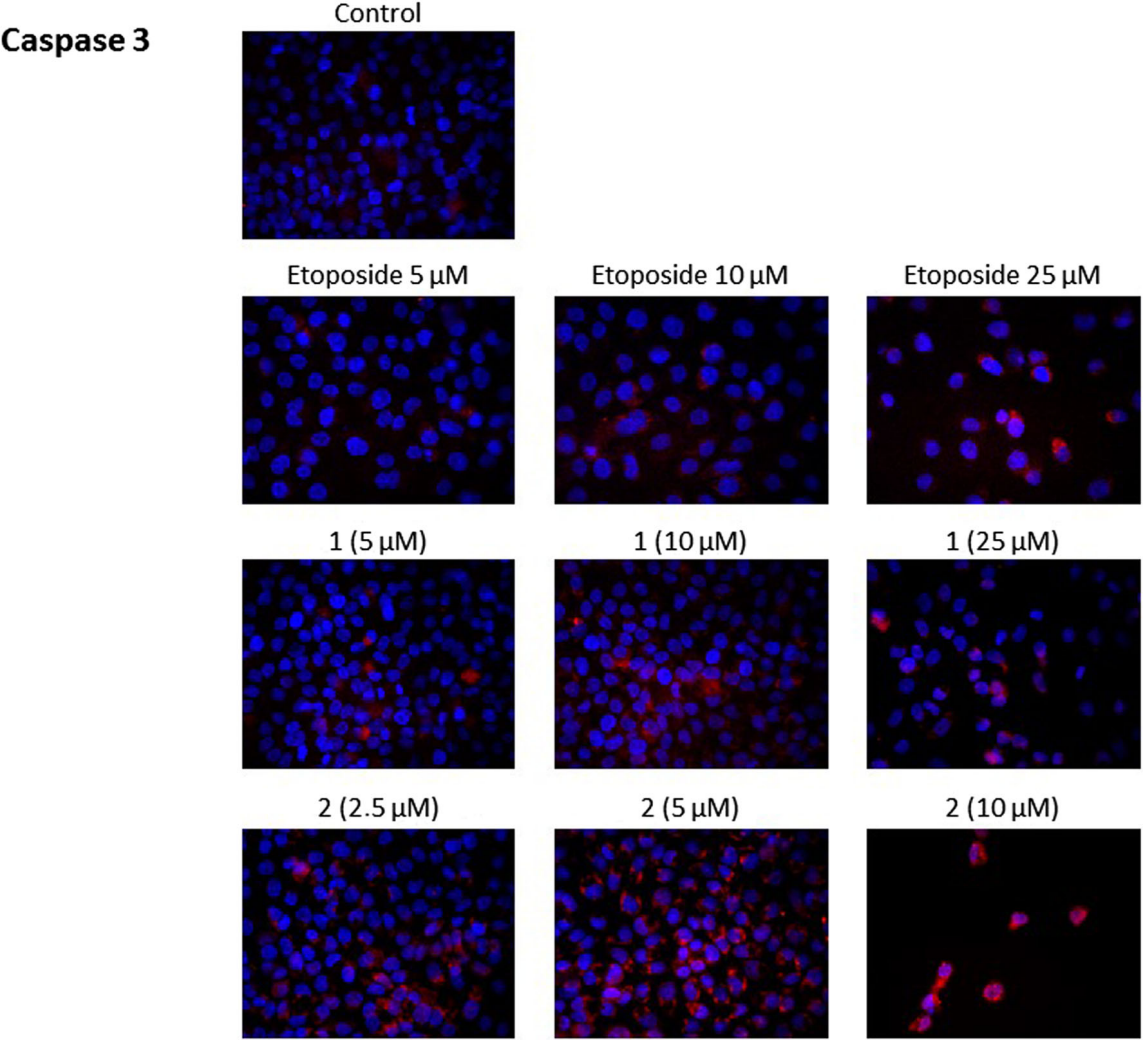
Immunofluorescence analysis of casapase 3 expression and translocation in human gastric AGS cells treated with different concentrations of the tested compounds (**1, 2**) and etoposide

The caspases are pivotal players in the best documented mechanism of cancer cell death. Initiator caspases activate executioner caspases that lead to demolishing of key structural proteins and activate other enzymes. DNA fragmentation and membrane blebbing are the morphological hallmarks of apoptosis [13]. The influence of the tested compounds on the expression of initiator and executioner caspases was demonstrated in Figs. 8 and 9. Cells were incubated for 24 h with etoposide and compound 1 at 5 μM, 10 μM and 25 μM concentrations. Compound 2 was very active, so doses were limited to 2.5 μM, 5 μM and 10 μM. All the compounds tested increased the expression of initiator caspase-9 in a dose dependent manner in gastric cancer cells in comparison with control (Fig. 8). The novel diisoquinoline derivatives led to higher expression of caspase-9 in gastric cancer cells. The stronger intensity of red fluorescence was detected in comparison with control and etoposide. Our study confirmed that mitochondrial apoptotic pathway plays a crucial role in the mechanism of cell death.

Caspase-3 is one of the executioner caspases and its expression was also increased in analyzed cancer cells after 24 h of incubation with the compounds tested (Fig. 9). The study confirmed that compound 2 was the most cytotoxic and the strongest activator of caspase-3. The significant effect was detected after treatment with compound **2** at 5 μM concentration compared to cells treated with compound **1** and etoposide in the same doses. The increase of concentration of compound 2 to 10 μM enhanced its cytotoxic properties, but the expression of caspase-3 was also very high.

### Novel diisoquinoline derivatives increased the expression of p53 in gastric cancer cells

P53 plays a key role as a regulator of the programmed cell death. It can modulate pivotal control points in death *receptors* signaling pathway and mitochondrial apoptotic pathwy. It can directly activate the transcription of genes resposible for promotion of apoptosis [14].

**Fig. 10:**
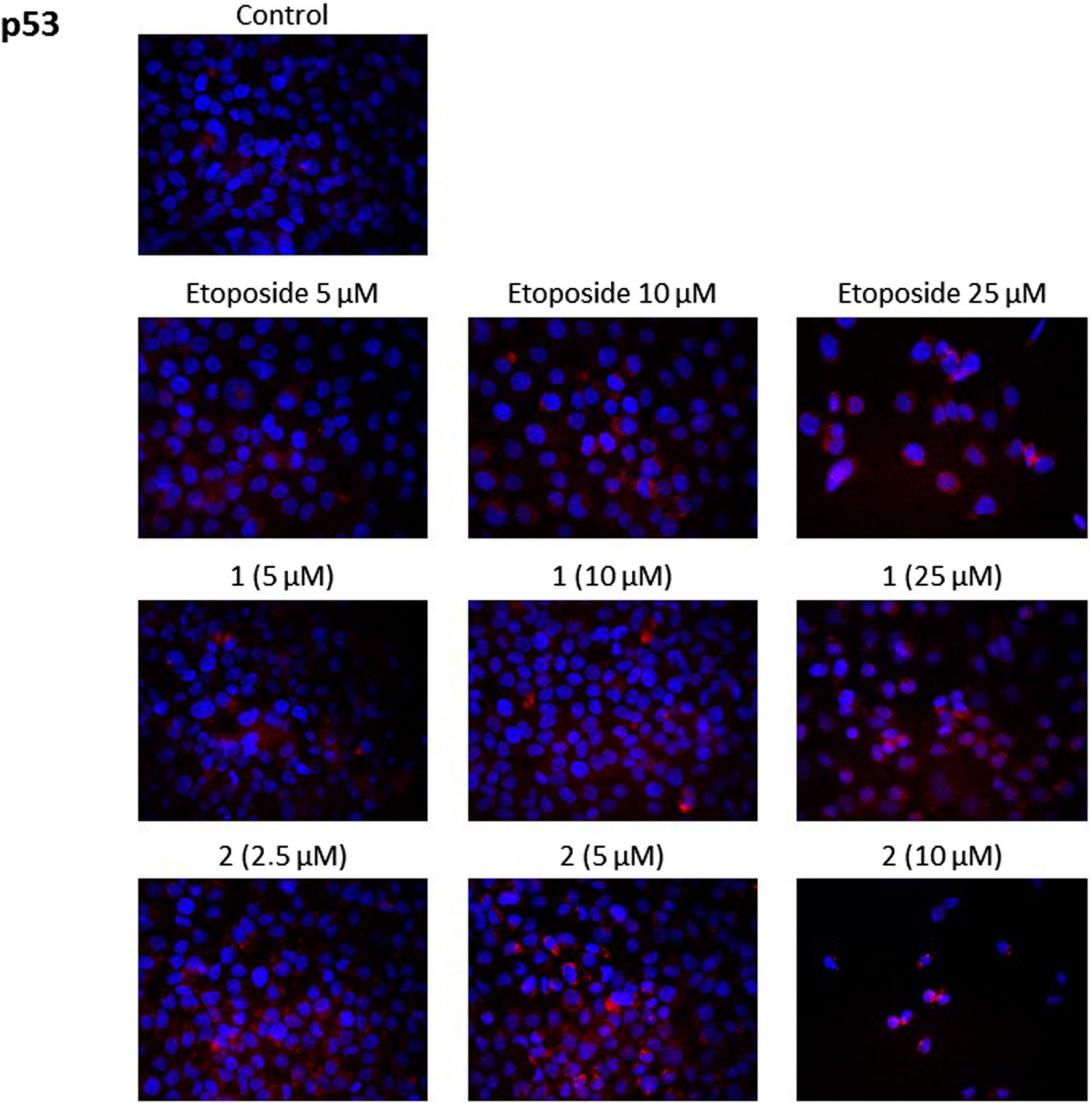
Immunofluorescence analysis of p53 expression and translocation in human gastric AGS cells treated with different concentrations of the tested compounds (**1, 2**) and etoposide

The expression of p53 protein was increased in a dose dependent manner after treatment with etoposide and the novel diisoquinoline derivatives **1** and **2**, compared to control. The strongest expression of p53 was observed after incubation with compound **2** (Fig. 10).

### Novel diisoquinoline derivatives inhibited AKT and ERK1/2 expression in gastric cancer cells

**Fig. 11:**
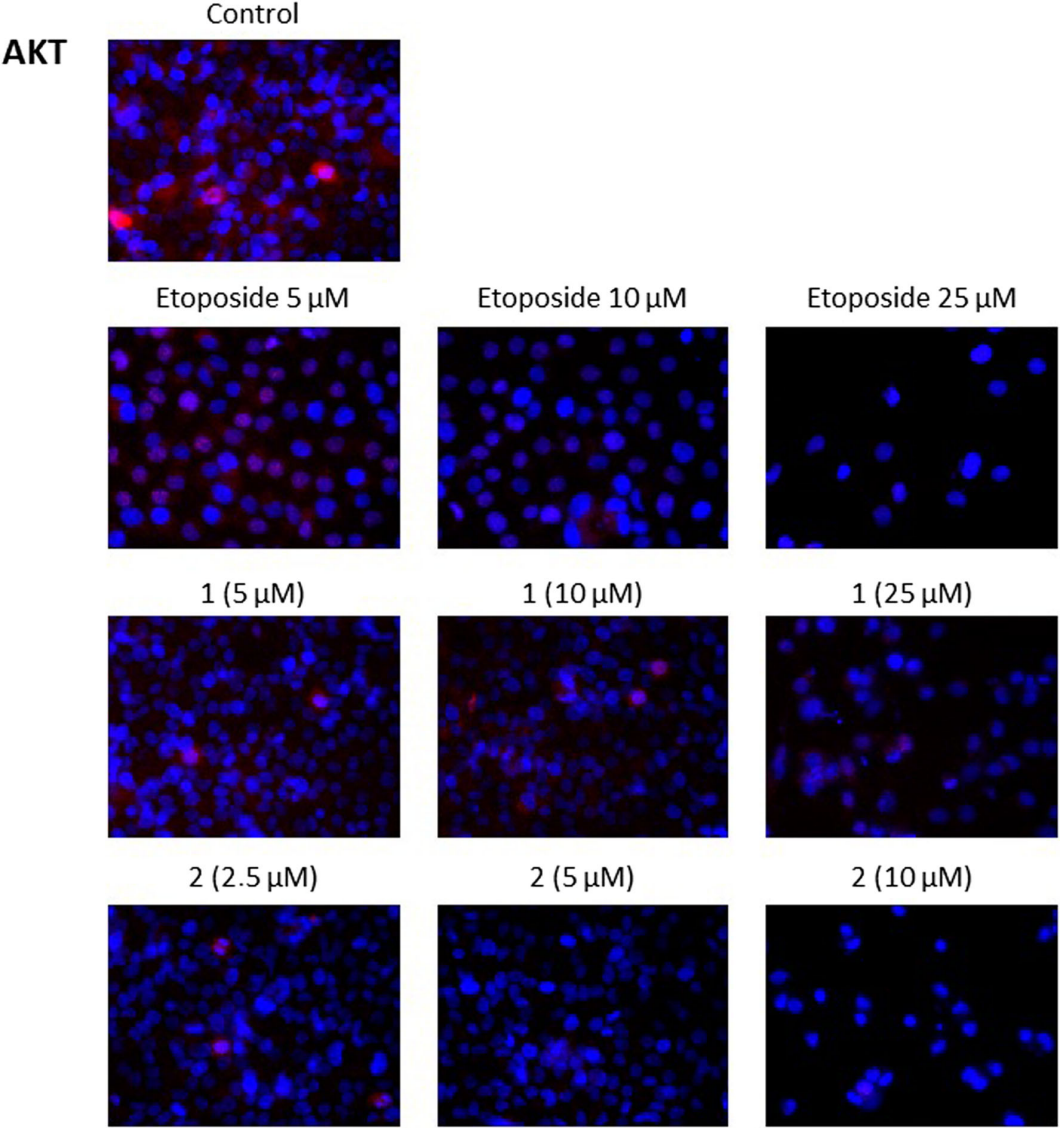
Immunofluorescence analysis of AKT expression in human gastric AGS cells treated with different concentrations of the tested compounds (**1, 2**) and etoposide

Finally, the effect of different concentrations of the tested compounds (**1**, **2)** and etoposide on the expression of AKT and ERK1/2 was analyzed in human gastric cancer cells. A large number of studies have suggested that one of the major functions of AKT/PKB is to promote growth factor-mediated cell survival and to block apoptosis. In our study, high intensity of red fluorescence, was observed in control cells without any agents (Fig. 11), thus confirming that AKT expression was higher in untreated cells. Cells exposed to various concentrations of compounds **1**, **2** and etoposide decreased the expression of AKT in all cases. The effect was enhanced with an increase in the doses from the lowest to the highest.

**Fig. 12:**
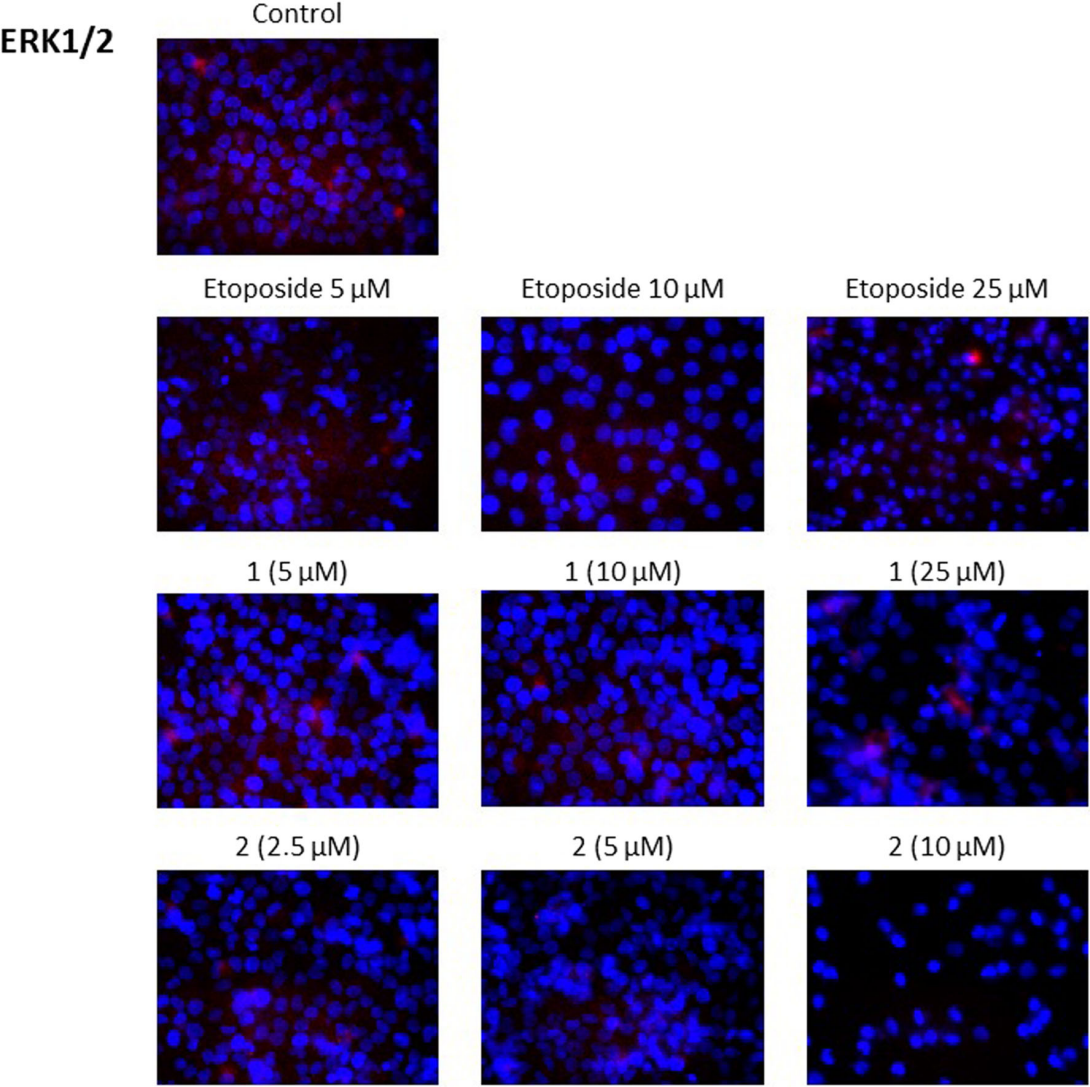
Immunofluorescence analysis of ERK1/2 expression in human gastric AGS cells treated with different concentrations of the tested compounds (**1, 2**) and etoposide

ERK activity can promote either intrinsic or extrinsic apoptotic pathways by the induction of mitochondrial cytochrome c release or caspase-8 activation [15]. All the compounds tested decreased the expression of ERK1/2 in comparison with untreated control (Fig. 12). Our research proved that the increase in the doses of etoposide and compound 1 from 5 μM to 25 μM decreased the intensity of red fluorescence. Compound 2 was the most effective inhibitor of kinase expression in the lower spectrum of doses (2.5 μM, 5 μM and 10 μM).

## Discussion

Despite progress in gastric cancer treatment, results are still unsatisfactory. The five-year survival rate of patients with gastric cancer is very low. Surgery, chemotherapy, radiation therapy, chemoradiation and targeted therapy represent five types of standard treatment in gastric cancer. Chemotherapeutic agents inhibit the growth of cancer cells by killing them or by inhibiting them from dividing. The 5-fluorouracil, mitomycin, cisplatin, etoposide and anthracyclins are active cytostatics used in gastric cancer treatment. Better results are observed after combined therapy, although higher toxicity is related with such a treatment.

**Fig. 13:**
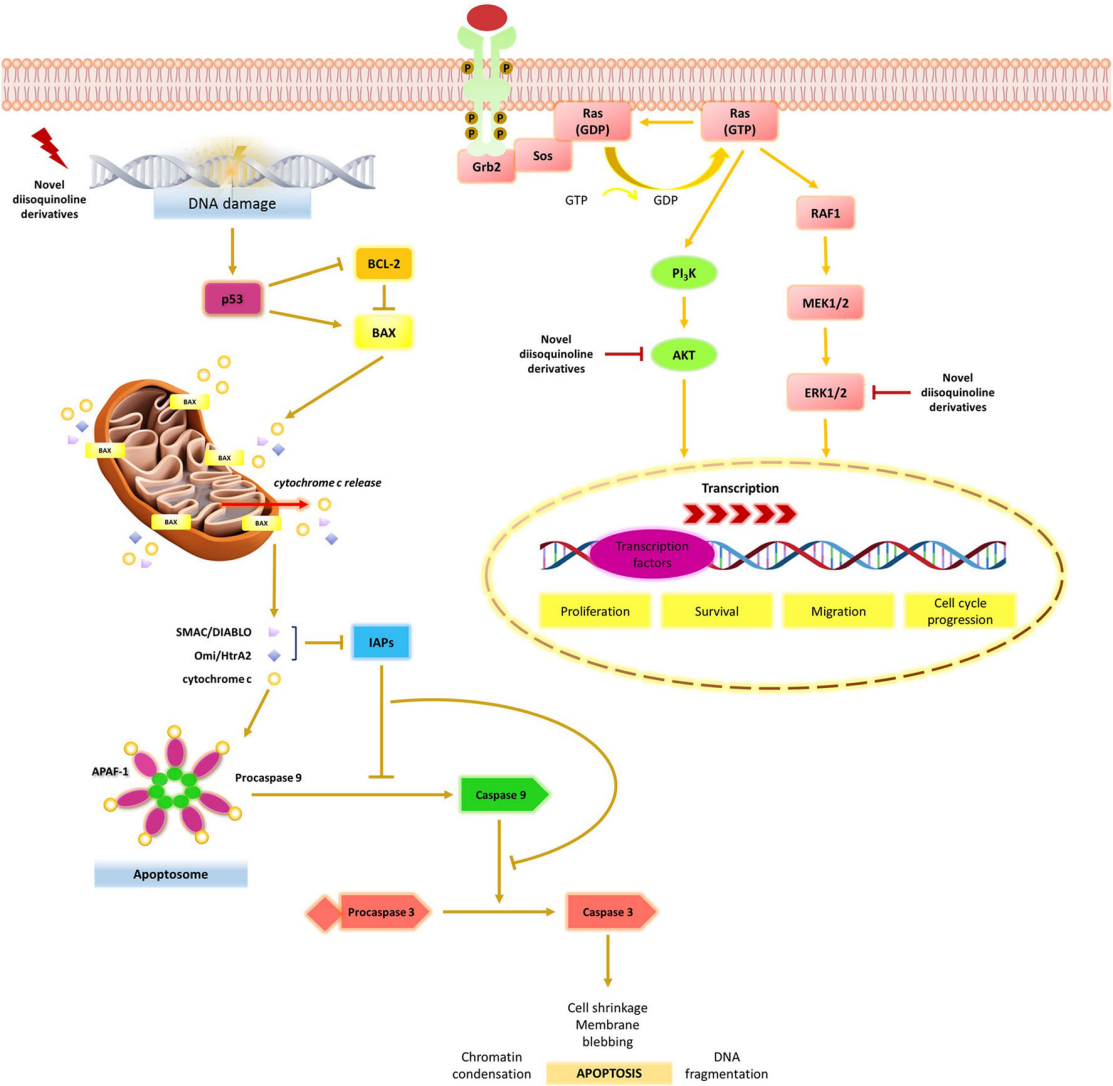
Mechanism of the tested compounds in gastric cancer cells

Our team is still looking for novel active drugs with high anticancer properties, but with better toxicological profile. Recently, we synthesized a group of novel diisoquinoline derivatives and we confirmed their anticancer potential in breast cancer cells [7]. The most promising agents were selected and their mechanism was analyzed in gastric cancer cells. The cytotoxic and antiproliferative effect of compounds **1** and **2** was associated with the induction of apoptosis (Fig. 13). Compound 2 was more effective in decreasing the viability of cancer cells and its IC_50_ value was 21 μM after 24 h of incubation and 6 μM after 48 h of incubation. The proapoptotic potential of compound 2 was almost four times stronger in comparison with etoposide after 48 h of incubation. Initiator caspase-9 and executioner caspase-3 were activated during the process of programmed cell death, which was demonstrated by confocal microscopy bioimaging. Our research also demonstrated that novel compounds led to the accumulation of cells in the G2/M phase of the cell cycle and strongly inhibited topoisomerase II. Their mechanism is similar to etoposide, which is a widely used drug for chemotherapy. Its mechanism is associated with the inhibition of topoisomerase II and cancer cells after treatment with etoposide accumulate at G2/M [11].

P53 is recognized as the guardian of the genome and plays a pivotal role in the regulation of cell cycle and DNA damage induced-apoptosis [11, 16]. It up-regulates the transcription of several pro-apoptotic members of the BCL-2 family, including BAX, PUMA, NOXA and BID, and inhibits the transcription of some anti-apoptotic members such as BCL-2 and BCL-XL [11, 17–20]. The inactivation of the TP53 tumour suppressor gene and mutation, which leads to the activation of the PI3K/AKT signaling pathway are well-described mechanisms in cancer cells to escape programmed cell death [21]. Confocal microscopy bio-imaging of p53 protein confirmed that the compounds **1**, **2** and etoposide increased the level of the analyzed protein in comparison with control. It proved that mitochondrial apoptotic pathway plays a crucial role in gastric cancer cell death and depends on p53 protein.

Many reports have proved that the AKT/PKB signaling pathway elucidates an important role in several cancers [22–25]. AKT/PKB has direct effects on the apoptosis pathway, e.g. targeting the pro-apoptotic Bcl-2 related protein, BAD. It also affects the transcriptional response to apoptotic stimuli, e.g.by affecting Forkhead factors and the activity of the p53 family [26]. Since the increased expression and activation of AKT are observed in many tumors, looking for novel AKT inhibitors is a potential strategy in anticancer treatment. Recent studies have found perifosine to be the most promising agent, inhibiting AKT translocation into cell membrane and thus stopping its activation. A preclinical study has proved that perifosine inhibits lung, prostate and breast cancer cell growth [27]. The synergistic action of perifosine with etoposide has also been well documented through activation of intrinsic and Fas-mediated extrinsic cell death pathways in human leukemia T cells [28]. Momota et al. showed that perifosine inhibits multiple signaling pathways in glial progenitors and cooperates with temozolomide to arrest cell proliferation in gliomas in vivo [29]. Our study proved that our novel compounds, especially compound 2 strongly decreased the level of p-AKT in AGS cells in comparison with control. The p-AKT inhibitory effect was much stronger than the reference etoposide.

ERK1/2 is an important subfamily of mitogen-activated protein kinases that control a wide spectrum of physiological processes. Their activation promotes cell survival under some circumstances, but on the other hand ERK1/2 has pro-apoptotic functions [30]. The proapoptotic function of the Ras /Raf /ERK pathway is well documented for apoptosis induced by DNA-damaging agents, such as etoposide [31–33], doxorubicin [31, 34–38], UV [31] and gamma irradiation [39]. ERK activity has been particularly implicated in cisplatin-mediated apoptosis in renal cells [33, 40–50].

Our immunofluorescence staining proved that etoposide inhibited the ERK1/2 expression in comparison with untreated gastric cancer cells. The strongest ERK1/2 inhibitory effect was observed after treatment with compound **2**. A similar effect was observed after treatment with trastuzumab, tamoxifen, doxorubicin, paclitaxel and cisplatin plus echistatin, which also blocked the extracellular signal-regulated kinase (ERK1/ERK2) and PI3K/AKT [51–53].

Our results suggest that novel diisoquinoline derivatives might be promising agents in gastric cancer treatment. Their mechanism is associated with p53-mediated apoptosis, accumulation of cells in the G2/M phase of the cell cycle and inhibition of topoisomerase II. The novel compounds are engaged in the inhibition of AKT and extracellular signal-regulated kinase (ERK1/ERK2). The strongest anticancer effect was observed after treatment with compound **2**, but further in vivo studies are also required.
